# miR-30a-5p attenuates hypoxia/reoxygenation-induced cardiomyocyte apoptosis by regulating PTEN protein expression and activating PI3K/Akt signaling pathway

**DOI:** 10.1186/s12872-024-03900-4

**Published:** 2024-05-05

**Authors:** Guoxin Liang, Chang Guo, Hongyue Tang, Mingming Zhang

**Affiliations:** 1 Department of Laboratory Medicine, Nanjing BenQ Medical Center, The Affiliated BenQ Hospital of Nanjing Medical University, Nanjing, Jiangsu 210000 China; 2 Graduate School, North China University of Science and Technology, Tangshan, Hebei 063210 China; 3 Clinical Medicine Research Center, Hebei Key Laboratory of Metabolic Diseases, Hebei General Hospital, 348#, Hepingxi Road, PO Box: 050051, Shijiazhuang, 050051 China; 4 School of Clinical Medicine, Graduate School of Hebei North College, Zhangjiakou, Hebei 075000 China

**Keywords:** Hypoxia/Reoxygenation (H/R), Apoptosis, microRNA (miRNA), Phosphatase and Tensin Homolog (PTEN), Transcriptomics

## Abstract

**Background:**

This study was designed to investigate the mechanism by which miR-30a-5p mediates cardiomyocyte apoptosis after acute myocardial infarction (AMI) induced by hypoxia/reoxygenation (H/R).

**Methods:**

Differentially expressed miRNAs were analyzed by RNA high-throughput sequencing in acute myocardial infarction (ST-elevation myocardial infarction) patients versus healthy individuals (controls). The H/R model was used to assess the regulatory mechanism of miRNAs in AMI. Lentivirus-associated vectors were used to overexpress or knock down miR-30a-5p in cellular models. The pathological mechanisms of miR-30a-5p regulating the development of acute myocardial infarction were serially explored by qPCR, bioinformatics, target gene prediction, dual luciferase, enzyme-linked immunosorbent assays (ELISAs) and Western blotting.

**Results:**

The results showed that the expression of miR-30a-5p was significantly increased in AMI patients and H9C2 cells. Hypoxia decreased cardiomyocyte survival over time, and reoxygenation further reduced cell survival. Bax and Phosphatase and tensin homolog (PTEN)were suppressed, while Bcl-2 was upregulated. Additionally, miR-30a-5p specifically targeted the PTEN gene. According to the GO and KEGG analyses, miR-30a-5p may participate in apoptosis by interacting with PTEN. The miR-30a-5p mimic decreased the expression of apoptosis-related proteins and the levels of the proinflammatory markers IL-1β, IL-6, and TNF-α by activating the PTEN/PI3K/Akt signaling pathway. Conversely, anti-miR-30a-5p treatment attenuated these effects. Additionally, silencing PTEN and anti-miR-30a-5p had opposite effects on H/R-induced cell apoptosis.

**Conclusions:**

miR-30a-5p plays a crucial role in cardiomyocyte apoptosis after hypoxia-induced acute myocardial infarction. Our findings provide translational evidence that miR-30a-5p is a novel potential therapeutic target for AMI.

**Supplementary Information:**

The online version contains supplementary material available at 10.1186/s12872-024-03900-4.

## Introduction

Acute myocardial infarction (AMI) has become a major threat to global health. Despite the improving prognosis of patients with myocardial infarction (MI), MI remains one of the leading causes of death and disability worldwide [1, 2]. Acute myocardial infarction (AMI) is defined as the apoptosis and necrosis of cardiomyocytes due to myocardial ischemia and subsequent myocardial hypoxia, ultimately leading to impaired cardiac function. Apoptosis usually occurs due to the dysregulation of pro-apoptotic and antiapoptotic genes in ischemic cardiac tissues [3]. It has been reported that oxidative stress, hypoxic injury, and ischemia–reperfusion induce apoptosis in the myocardial infarction zone, which can lead to the loss of cardiomyocytes, aggravate cardiac insufficiency, or even cause heart failure [4]. Therefore, exploring the mechanisms by which hypoxia regulates cardiomyocyte apoptosis after myocardial infarction (post-MI) may provide an important molecular basis for the prevention and treatment of MI.

In recent decades, several miRNAs have been shown to be involved in cardiomyocyte apoptosis during the progression of AMI. A large number of microRNAs show significant changes during the pathological process of MI [5, 6]. In addition, many abnormally altered microRNAs can be used as biomarkers for MI. It was found that miR-30a-5p expression was significantly upregulated in blood samples from patients with congestive heart failure [7]. In addition, significant upregulation of miR-30a-5p expression can be detected in cardiac hypertrophy and myocardial fibrosis [8]. MicroRNAs (miRNAs) are noncoding RNAs 21–23 nucleotides in length that play important regulatory roles in plants and animals by modulating the expression of target mRNAs for cleavage or translational repression at the posttranscriptional level [9, 10]. Phosphatase and tensin homolog (PTEN) is a potential target gene for miR-30a-5p. PTEN is a well-known tumor suppressor [11]. In addition, a growing number of studies have shown that PTEN plays an important role in cardiovascular diseases, including MI. For example, PTEN promotes the expression of the cytokines TNF-α, IL-1β, IL-6, and IL-18 and mediates vascular inflammation and apoptosis [12]. In mice suffering from acute myocardial infarction, PTEN expression is significantly increased, which contributes to cardiomyocyte injury. High PTEN expression exacerbates left ventricular remodeling after MI [13, 14]. In addition, PTEN negatively regulates the phosphorylation of phosphatidyl inositol 3-kinase (PI3K) and protein kinase B (AKT) to modulate programmed cell necrosis [15]. However, the role and potential mechanism of miR-30a-5p in cardiomyocyte apoptosis in the context of acute myocardial infarction are unclear and unknown.

In the present study, we found that miR-30a-5p expression was significantly upregulated in ischemic hearts and H_2_O_2_-treated cardiomyocytes. Specifically, miR-30a-5p inhibited cardiomyocyte apoptosis and myocardial dysfunction during AMI progression by directly targeting PTEN. Our findings contribute to the understanding of the complex mechanisms of AMI progression and provide new therapeutic targets for AMI treatment.

## Materials and methods

### Study subjects

Patients with AMI (age: 20 – 65 years) were enrolled in the study. The Ethics Committee of Hebei General Hospital approved the study protocol (approved No: 202104). The inclusion criteria for patients were as follows: discharge from AMI; first episode of AMI; and coronary angiography. The exclusion criteria were as follows: previous history of definite myocardial infarction; history of cardiac insufficiency; history of combined hepatic, pulmonary, and renal insufficiency; and incomplete clinical data. Healthy control participants were recruited among individuals undergoing wellness. Healthy control participants were recruited among individuals undergoing a wellness examination. Informed consent was obtained from all participants. This study included 245 individuals, 155 AMI patients and 90 healthy individuals.

### Bioinformatics analysis

The GEO datasets (https://www.ncbi.nlm.nih.gov/geo/) GSE230165, GSE48060, and GSE66360 were selected to identify the genes differentially expressed after MI. The limma package (version 3.26) was used to screen the differentially expressed miRNAs, and miRNAs with *P* < 0.05 and log2-fold change (FC) > 1 or log2FC < 1 are shown in the heatmap [16]. The target genes of miR-30a-5p were predicted based on the TargetScan and miRWalk databases, and a dual-luciferase assay was performed to assess whether miR-30a-5p directly binds to PTEN. mRNA‒miRNA interaction networks were constructed by employing Cytoscape 3.7.1 (https://cytoscape.org/). Significant correlations between the miRNA-mediated signaling pathways were investigated using the Gene Ontology (GO) and KEGG databases.

### Culture, modeling, and transfection of H9c2 cardiomyocytes

H9c2 cardiomyocytes were purchased from the Cell Bank of the Shanghai Institute of Cell Biology (Shanghai, China). The cells were grown in Dulbecco's modified Eagle's medium (DMEM, Logan, HyClone, UT, USA) with 10% fetal bovine serum (FBS, Gibco, Grand Island, NY, USA) in an incubator with 5% CO2 at 37 °C. The medium varied every two days. To establish the hypoxic model, the cells were placed in an incubator with 5% CO2 for 12 h before being injected with 99.9% N2.The monitor assessed the volume fraction of O2, and a value of less than 1% indicated anoxia.The H9c2 cells were then incubated in reoxygenation (95% air, 5% CO2) for 12 h.Transient transfection of cardiomyocytes with miR-30a-5p mimics, NC microRNA, anti-miR-30a-5p, PTEN-specific small interfering RNA (siRNA) or NC siRNA (100 nM/10^5^ cells) was performed using Lipofectamine 3000 reagent.

### Quantitative real-time polymerase chain reaction (qRT‒PCR)

Total RNA was extracted from H9c2 cells via TRIzol reagent and quantified by spectrophotometry. For miRNA detection, 1 μg of total RNA was reverse transcribed in the presence of a stem ring and amplified using a Bulge-Loop TM miRNA qRT‒PCR Starter Kit (C10211–2, RiboBio Biotechnology, Guangzhou, China). The most stable expression, U6, was the endogenous control, and the test was repeated three times. The Ct value was calculated and analyzed by the 2^−△△Ct^ method, and the relative expression level of miR-30a-5p was determined.

### Western blot analysis

H9C2 cardiomyocytes in the logarithmic growth phase were harvested. After digestion, dilution, transfection, grouping, and other procedures, the cells were washed with PBS solution. A lysis solution was added, the samples were centrifuged for 15 min, and a BCA kit was used to determine the total protein concentration. Thirty micrograms of protein were added to 5 × sampling buffer and placed in a hot water bath for 5 min. This causes protein denaturation. After cooling to room temperature, the samples were loaded onto a 10% SDS‒PAGE gel for spiking. After blocking with 5% skim milk, the membranes were incubated with the primary antibodies listed in Table S1. ImageJ software was used to quantify the gray values of the protein fragments.

### MTT assay for cell viability

Briefly, H9c2 cardiomyocytes were inoculated at a density of 5 × 10^4^ cells/mL in 96-well plates and incubated with MTT (5 mg/mL, Sigma‒Aldrich) for 4 h. The medium was then discarded by centrifugation, and 150 μL of DMSO was added to lyse the cells to dissolve the methanol. Cell viability was quantified by measuring the absorbance (optical density) at 490 nm on a microplate reader, and the results are expressed as the fold change relative to that of the vehicle control treatment (PBS), as previously described.

### Dual luciferase activity assays

The target genes of miR-30a-5p were predicted based on the TargetScan and miRWalk databases, and a dual-luciferase assay was performed to assess whether miR-30a-5p directly binds to PTEN. The partial 3′-UTR of PTEN in rat genomic DNA was amplified by PCR, cloned, and inserted into the pSI-Check2 vector to generate a wild-type (Wt) reporter gene. A mutant (Mut) reporter gene was generated by targeted mutagenesis of the miR-30a-5p seed-matched region. Plasmid constructs and dual luciferase kits were purchased from Hanbio Biotechnology. Following the transfection procedure, the pSI-Check2-PTEN-3′UTR (Wt or Mut) was incubated with vector (control), miRNA negative control oligonucleotide (miRNA -Scr) or miRNA negative control oligonucleotide (miRNA-Scr) using Lipofectamine™ 3000 (L3000001, Thermo Fisher Scientific, Waltham, MA, USA). miRNA NC) or miR-30a-5p mimic was cotransfected into H9c2 cells. Cell lysates were measured 24 h after transfection, and dual-luciferase reporter activity was assayed using a kit (Hambro Bio, Wuhan, China) and a multimode microplate reader (Promega GloMax-Muti®, Promega, Madison, WI, USA).

### ELISA

H9C2 cardiomyocytes in the logarithmic growth phase were collected, digested, diluted, transfected, and grouped. The cells were suspended in PBS, and the cell membrane was disrupted by repeated freeze-thawing 3 times. Three replicate wells were set up, and the operations were performed according to the instructions of the TNF-α, IL-6, and IL-1β kits. The absorbance of each group was detected at a wavelength of 450 nm.

### Statistical analysis

Analysis was performed with GraphPad Prism 9.3, and the results are presented as the mean ± SD. The Shapiro‒Wilk test was performed to determine the distribution of the data. For normally distributed variables, Student's t-test or analysis of variance (ANOVA) was used. Variables that were not normally distributed were analyzed using the Mann‒Whitney or Kruskal‒Wallis test. The statistical significance level was set at *p* < 0.05.

## Results

### Expression levels of miR-30a-5p were abnormally increased after H/R and MI

**Fig. 1:**
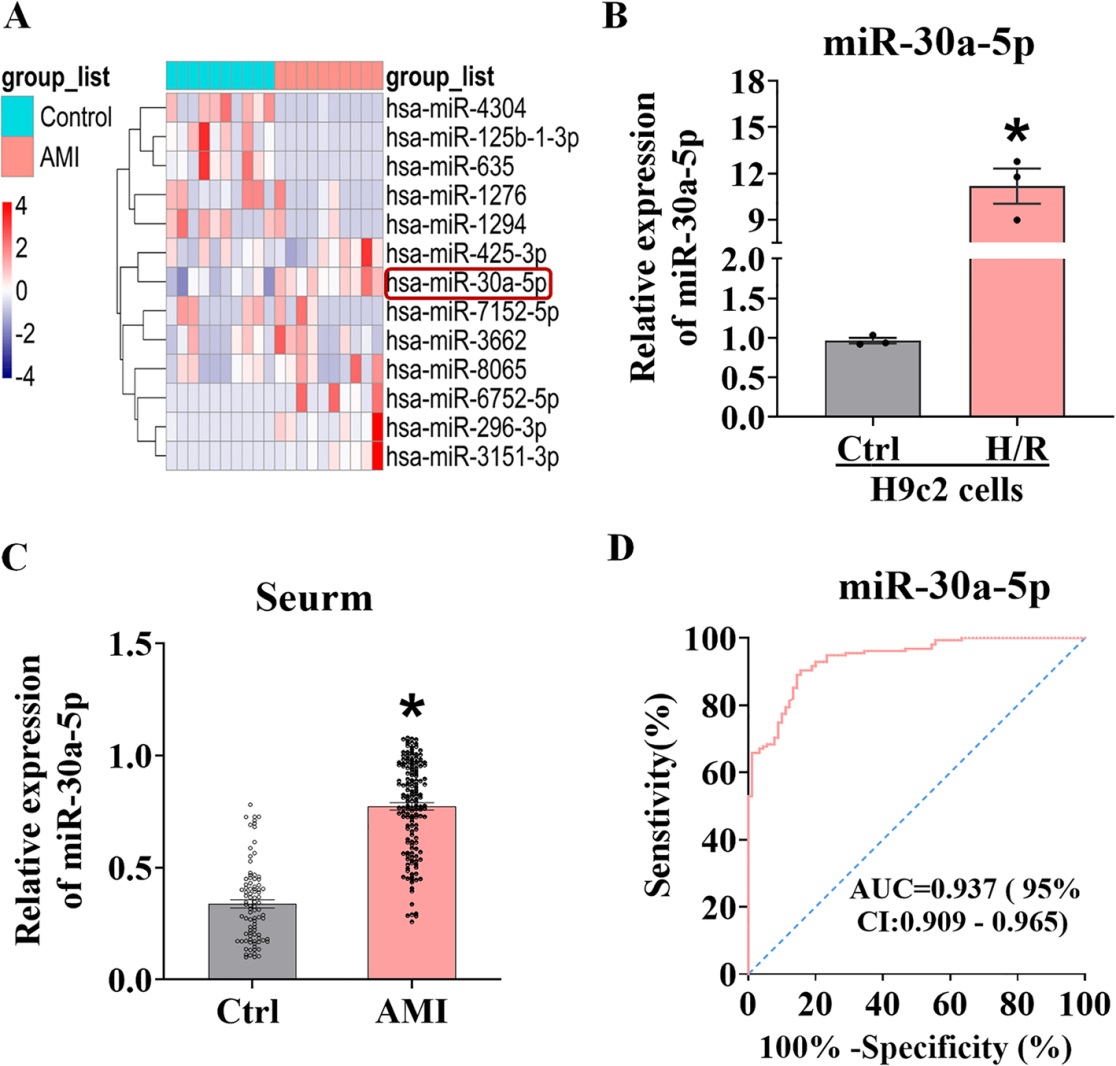
Elevated expression levels of miR-30a-5p and its diagnostic value. **A** Heatmap of miRNAs with differential expression. One dysregulated miRNA is represented by each row, and one sample is represented by each column. **B** Comparison of the relative expression of miR-30a-5p in H9c2 cardiomyocytes after H/R treatment and in cells from the control group (*n* = 3); *P < 0.05 vs. the control group. **C** The serum levels of miR-30a-5p were greater in patients with AMI (*n* = 155) than in healthy controls (*n* = 90); *P < 0.05 vs. the control group. **D** ROC analysis of miR-30a-5p to discriminate AMI patients from healthy control subjects

Analysis of the published microarray dataset GSE230165 revealed that miR-30a-5p expression was significantly upregulated in the blood samples of MI patients (Fig. 1A). qRT‒PCR showed that, compared with that in the control groups, miR-30a‒5p expression was significantly upregulated in both H/R-treated H9c2 cell lines (Fig. 1B). Further analysis of the clinical samples by qRT‒PCR revealed that the expression of miR-30a-5p was significantly greater in the serum of AMI patients than in that of sex- and age-matched normal control participants (Fig. 1C). Receiver operating characteristic (ROC) curve analysis indicated that the area under the curve (AUC) was 0.937 (95% CI: 0.801–0.912; cutoff = 0.468), with 84.4% sensitivity and 90.3% specificity in discriminating AMI patients from normal control participants (Fig. 1D).

### PTEN is a target gene of miR-30a-5p

**Fig. 2:**
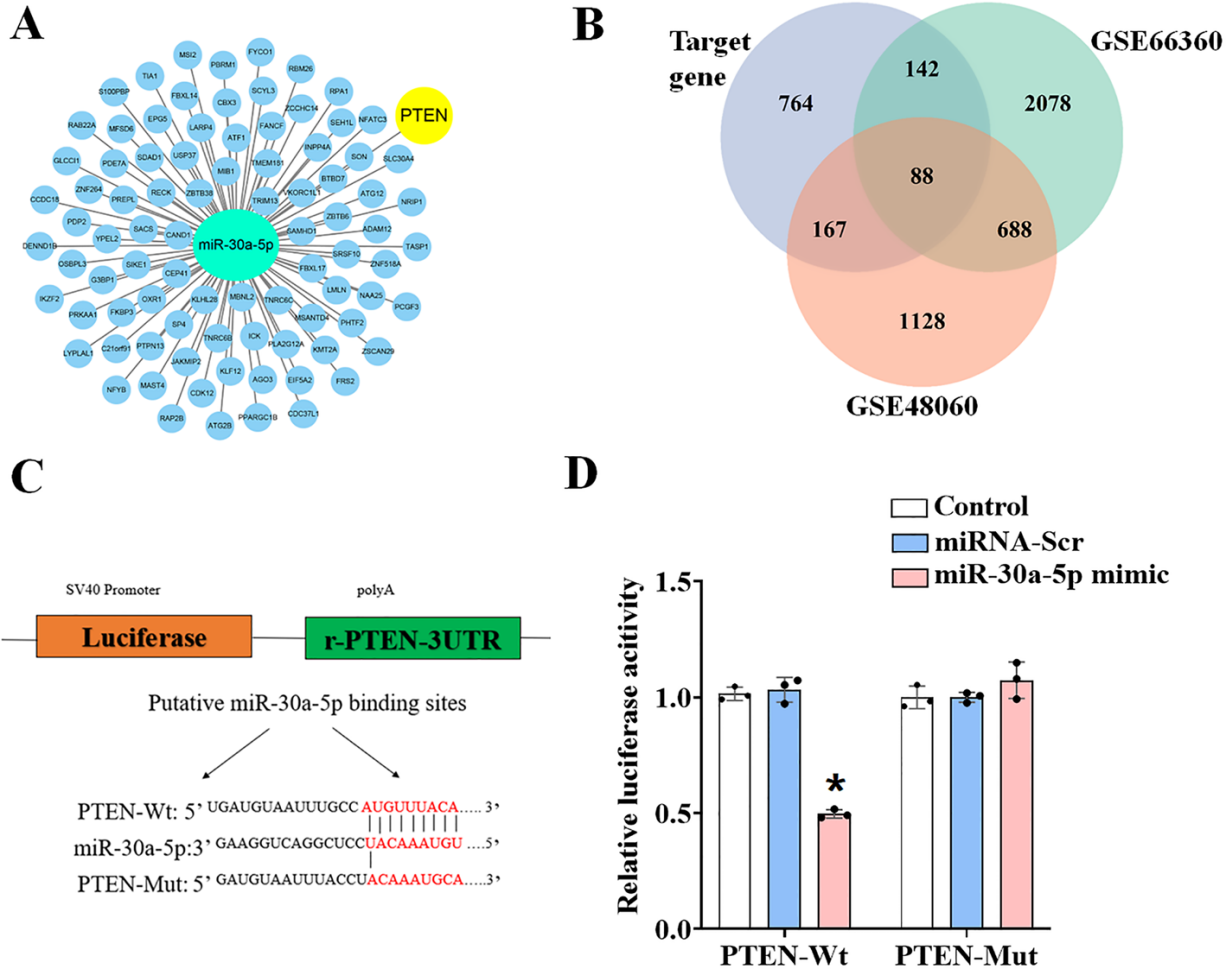
Bioinformatics analysis was used to predict miR-30a-5p target genes and validate target genes. **A** A network diagram showing the bioinformatics prediction of miR-30a-5p target genes. **B** Venn diagram of the intersecting genes between DEGs and target genes of miR-30-5p. **C** Potential binding sequences of PTEN and miR-30a-5p 3' noncoding regions. **D** The PTEN-Wt or PTEN-Mut reporter vector was cotransfected into H9c2 cells with vehicle (control), miR-30a-5p mimic, or negative control (miRNA-Scr). The normalized luciferase activity of the control group was established as the relative luciferase activity. The data are expressed as the mean ± standardized value of the mean (*n* = 3)

Microarray screening and bioinformatic analysis of potential target genes of the predicted miRNA targets via the miRDB and TargetScan databases revealed that PTEN was a potential target gene of miR-30a-5p (Fig. 2A, B). We therefore used a dual luciferase reporter to verify the predictions in H9c2 cells. The miR-30a-5p binding sequences in the 3′ UTR of WT PTEN mRNA (PTEN-Wt) or its mutant (PTEN-Mut) were subcloned downstream of the firefly luciferase reporter gene in a pSI-Check2 vector. We observed that the miR-30a-5p mimic significantly inhibited the luciferase activity of the PTEN 3′-UTR reporter but did not affect the luciferase activity of the PTEN 3′-UTR reporter with mutated miR-30a-5p binding sites. Furthermore, there was no change in luciferase activity when a negative control miRNA was cotransfected with either the WT or the reporter construct (Fig. 2C, D). These results indicate that PTEN is a target of miR-30a-5p.

### Anti-miR-30a-5p upregulates PTEN and promotes apoptosis in cardiomyocytes

**Fig. 3:**
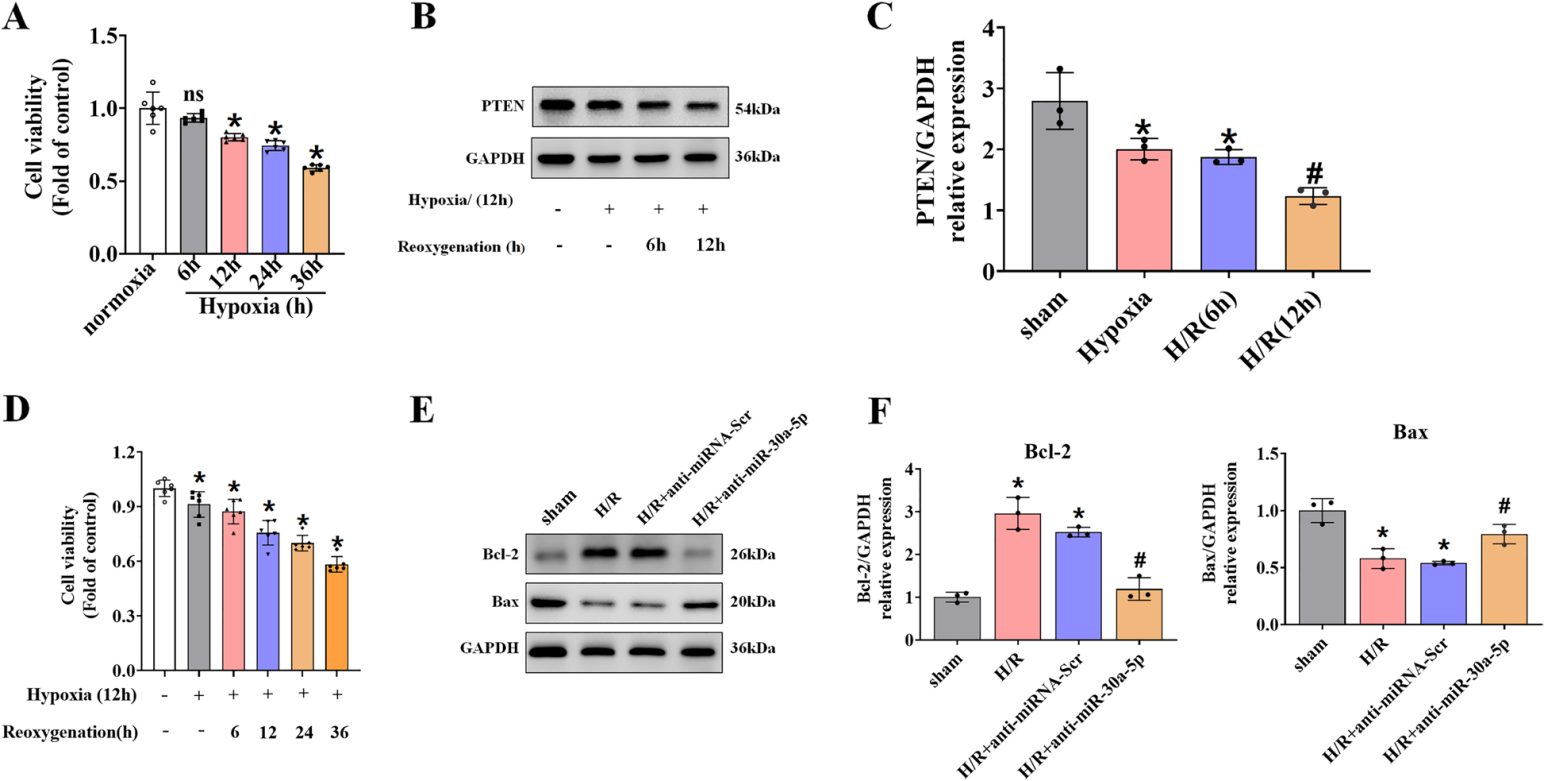
Upregulation of miR-30a-5p promotes cardiomyocyte apoptosis. **A** Cell viability determined after normoxic or hypoxic incubation. **B**, **C** Representative Western blot images of PTEN and densitometric quantification of PTEN/GAPDH. **D** Cell viability as measured after normoxic culture, hypoxia alone (12 h), or reoxygenation after 6–36 h of hypoxia. **E**, **F** Representative bands were obtained from c-caspase-3, Bax, and Bcl-2 experiments and densitometric quantification. The results are presented as the mean ± SD. Compared with the sham group, *p < 0.05; compared with the miRNA-Scr group, #*p* < 0.05. *n* = 3 for each group

We used the MTT assay to assess H9C2 cardiomyocyte viability during hypoxia treatment to further analyze the impact of miR-30a-5p on hypoxia-reoxygenation-induced apoptosis. Hypoxia reduced cardiomyocyte viability over time, with a significant decrease starting 12 h after hypoxia treatment. The effect of different durations of reoxygenation on cardiomyocyte viability was then examined by exposing the cardiomyocytes to hypoxia for 12 h (Fig. 3A, D). With increasing duration of reoxygenation, hypoxia-induced cell death decreased even further. Even after 6 h of reoxygenation, there was a significant decrease in cardiomyocyte viability compared to 12 h of hypoxia exposure alone. Subsequent reoxygenation after hypoxic exposure resulted in a gradual reduction in PTEN protein expression in cardiomyocytes reoxygenated at the beginning of the 12-h reoxygenation period compared to normoxic culture conditions (Fig. 3B, C). Western blot analysis revealed that anti-miR-30a-5p significantly increased Bax and decreased Bcl-2 levels (Fig. 3E, F).

### miR-30a-5p attenuates cardiomyocyte injury by negatively regulating PTEN expression and activating the PI3K/Akt signaling pathway

**Fig. 4:**
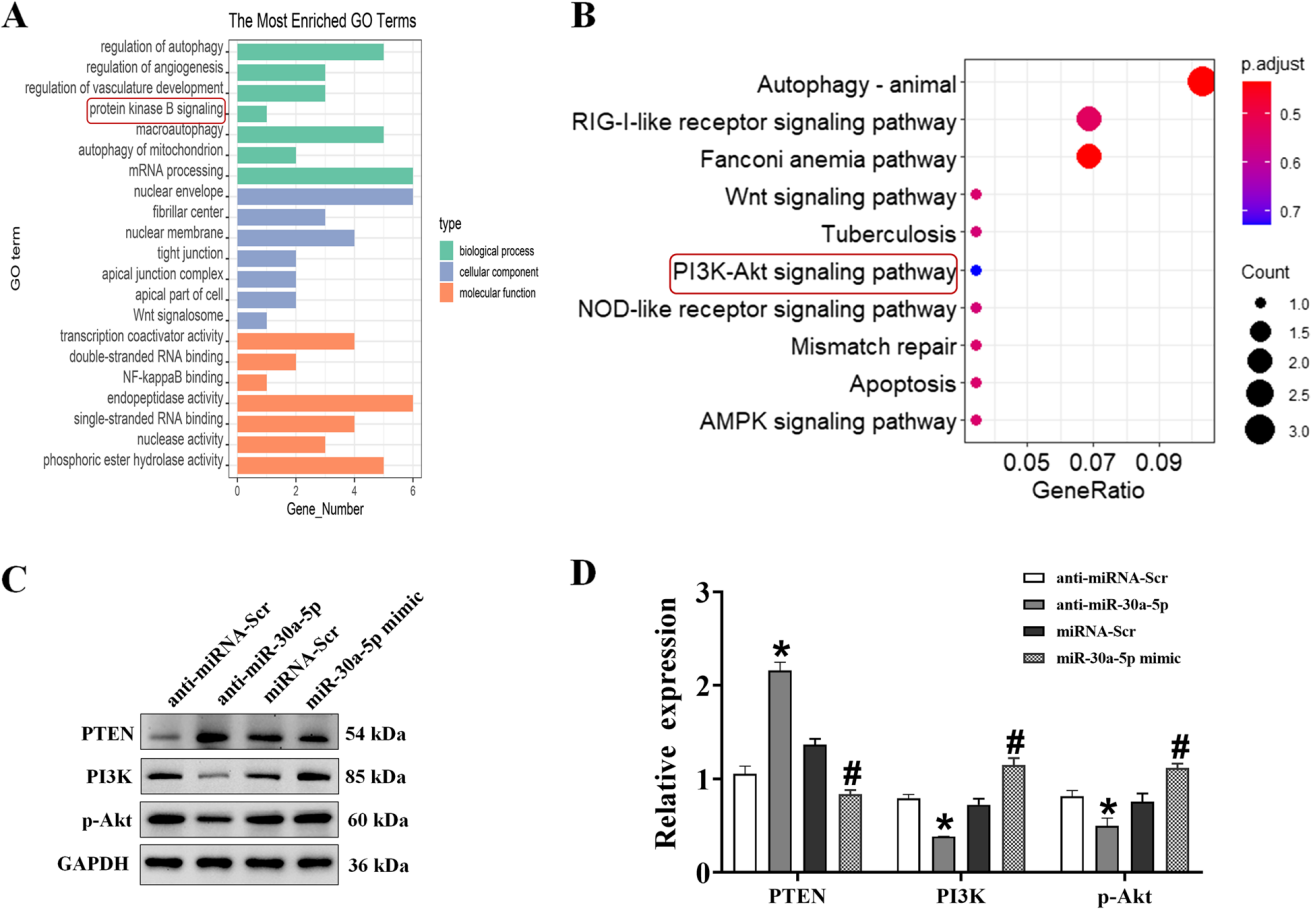
miR-30a-5p affects hypoxia/reoxygenation (H/R) progression by targeting PTEN to activate the PI3K/Akt signaling axis. **A**, **B** GO and KEGG enrichment analysis of common genes. **C** Representative bands showing PTEN, PI3K, and p-AKT expression in H9C2 cells from the miRNA-Scr, miR-30a-5p mimic, anti-miRNA-Scr, and anti-miR-30a-5p groups (*n* = 3). **D** Densitometric quantification of the PTEN/GAPDH, PI3K/GAPDH, and p-AKT/GAPDH ratios. *P < 0.05 compared with the miRNA-Scr group

Kyoto Encyclopedia of Genes and Genomes (KEGG) and Gene Ontology (GO) pathway analyses of the predicted target genes revealed that miR-30a-5p is involved in AMI and plays a vital role in apoptosis. The TargetScan and miRWalk databases revealed that miR-30a-5p may regulate PTEN. The PI3K-Akt signaling pathway was one of the most enriched Gene Ontology categories (Fig. 4A, B). Therefore, we selected the PI3K-Akt signaling pathway and apoptosis pathway for further experiments. Next, we explored potential downstream effectors of miR-30a-5p activation in PTEN-mediated H/R injury. We detected the expression of PI3K and Akt, the main effector molecules in the PI3K-Akt pathway, and found that in H9c2 cardiomyocytes treated with the miR-30a-5p mimic, the phosphorylation of AKT at Thr308 and Ser473 was dramatically increased, while the opposite effect was observed for anti-miR-30a-5p (Fig. 4C, D).

### miR-30a-5p regulates the PTEN/PI3K/Akt signaling pathway, thereby modifying H/R-induced cardiomyocyte damage

**Fig. 5:**
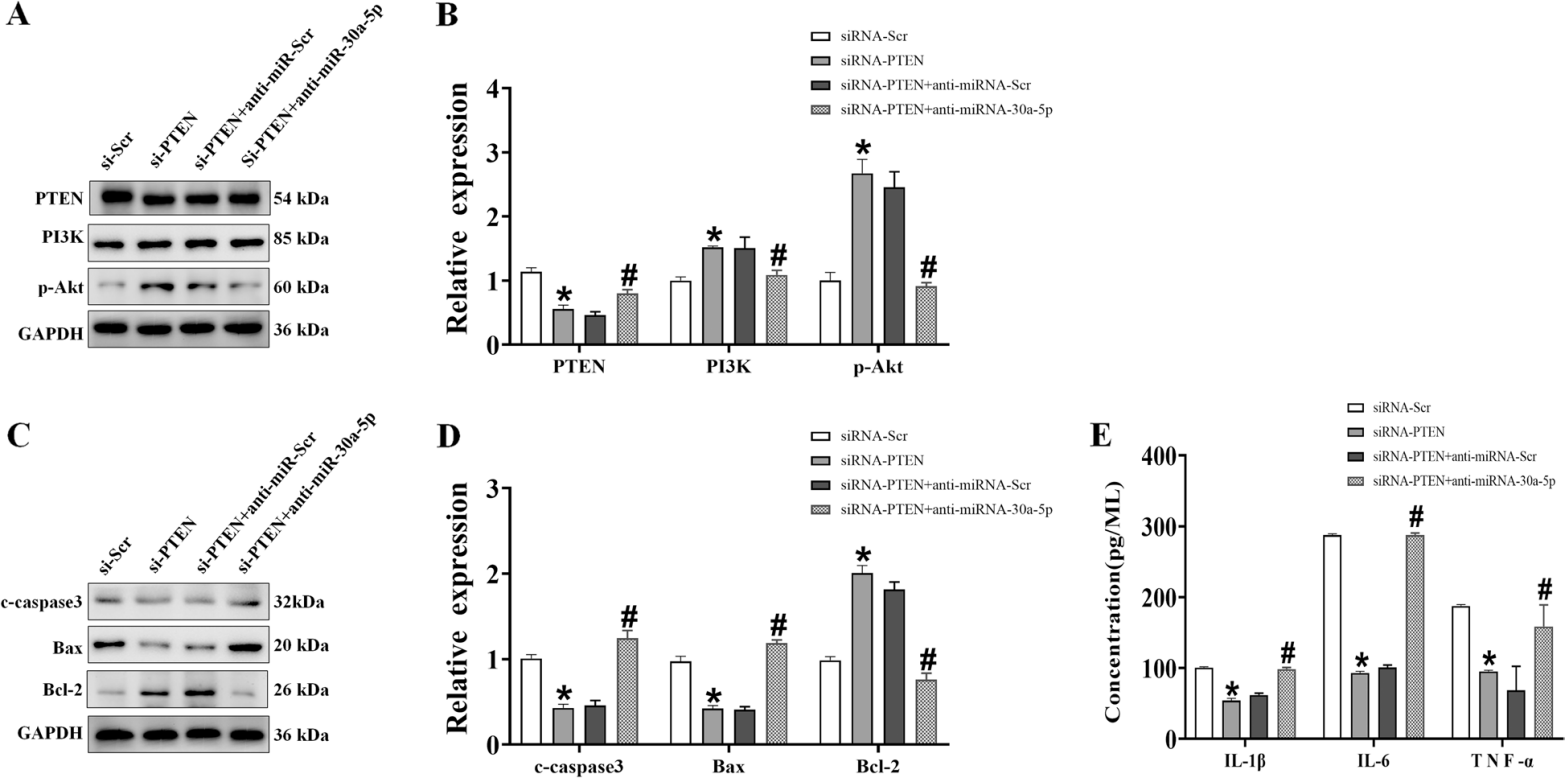
miR-30a-5p promotes hypoxia/hypoxia-induced inflammation and apoptosis by regulating the PTEN/PI3K/Akt axis. **A** Representative western blot bands of PTEN, PI3K, and p-Akt. **B** The protein levels of PTEN, PI3K, and p-Akt in cardiomyocytes were determined using WB analysis. **C** Representative western blot bands of c-caspase-3, Bax, and Bcl-2. **D** Protein levels of c-caspase-3, Bax, and Bcl-2 in cardiomyocytes were determined using WB analysis. **E** The levels of cytokines such as IL-1β, IL-6, and TNF-α were detected by ELISA (*n* = 3). ^*^P < 0.05 vs the si-NC group, ^#^*P* < 0.05 vs the anti-miR-30a-5p + si-PTEN group

To determine whether PTEN/PI3K/Akt is involved in cardiomyocyte damage, we administered PTEN inhibitor of bpV(pic). Western blot analyses showed that silencing PTEN resulted in increasing the levels of PI3K and p-Akt and decreasing levels of PTEN (Fig. 5A, B). We detected apoptosis-related proteins to determine whether cardiomyocyte damage was blocked by the increase of miR-30a-5p. Compared to the si-NC group, the levels of Bcl-2 (an antiapoptotic factor) was significantly elevated, and the levels of Bax (a proapoptotic factor) was reduced in the H/R group. However, these changes were mitigated by anti-miR-30a-5p mimic administration (Fig. 5C, D). Finally, the levels of the proinflammatory markers IL-1β, IL-6, and TNF-α were significantly reduced, which was exacerbated by silencing PTEN expression, whereas anti-miR-30a-5p reversed these effects and elevated the levels of inflammatory factors (Fig. 5E). These results suggest that anti-miR-30a-5p decreases apoptosis in H9c2 cells after H/R treatment.

## Discussion

Myocardial infarction is one of the most dangerous cardiovascular conditions. The irreversibility of post-infarction myocytes seriously threatens patients' lives and health, as it lowers ventricular contractility and eventually leads to heart failure [17]. Additionally, the H/R-induced apoptotic response of myocytes exacerbates myocardial damage. Therefore, it is crucial to investigate the molecular mechanisms underlying cardiomyocyte apoptosis to develop new therapeutic options for cardiovascular disease (CVD) [18]. Several studies have suggested that the upregulation of genes such as the Bcl-2 family, Bax, nuclear factor-κB, and p53 is associated with increased apoptosis in various tissues following hypoxia/reoxygenation (H/R) [19].

Additionally, current research on miRNAs aims to mitigate cardiomyocyte apoptosis during myocardial ischemia/reperfusion (I/R). miRNA-7a/b regulate the levels of key molecules associated with cardiomyocyte apoptosis during myocardial ischemia/reperfusion (I/R) [20]. Inhibition of miRNA-30 through the regulation of cysteine sulfide-γ-lyase expression is used to prevent ischemic damage to the heart [21]. Additionally, early reports have shown significant upregulation of miR-30b-5p expression in the human myocardial cell line AC16 treated with hypoxia. Downregulation of miR-30b-5p can effectively alleviate hypoxia-induced cardiomyocyte damage. Aven is a potential target gene of miR-30b-5p, and its downregulation can partially reverse the effects of miR-30b-5p knockdown in AC16 cells under hypoxic conditions [22]. The mechanism of miR-30a-5p in H9c2 cells subjected to H/R was also validated. High expression of miR-30a-5p was observed in both H/R cell models and AMI patients. Evidence has shown that miR-30a-5p in peripheral blood has diagnostic value for AMI. Furthermore, the data confirmed that the downregulation of PTEN is associated with the inhibition of apoptosis in H9c2 cells subjected to H/R. The expression of the apoptotic proteins Bax and Bcl-2 was evaluated using Western blot analysis. In H9c2 cells subjected to H/R, downregulation of miR-30a-3p inhibited Bcl-2 expression and increased Bax expression.

This study utilized high-throughput technology to investigate differentially expressed miRNAs in the serum of AMI patients. We further predicted corresponding downstream target genes and pathways using bioinformatics. MiRNAs exert their effects by binding to the 3'-UTR of target genes. Through the use of luciferase reporter gene detection and bioinformatics analysis databases, we identified the 3'-UTR of miR-30a-5p that binds to PTEN. PTEN is a tumor suppressor gene with dual-specific phosphatase activity. It regulates cell proliferation and apoptosis by inhibiting the PI3K-Akt signaling pathway through dephosphorylated phosphatidylinositol.

Previous studies have shown that various miRNAs, such as miR-21, miR-22, and miR-144-5p, can target PTEN and regulate the Akt pathway, which affects cell proliferation, invasion, and apoptosis and plays an important role in the occurrence and development of tumors [23, 24]. uric acid inhibits the expression of miR-21 in a concentration- and time-dependent manner. They also found that miR-21 can increase myocardial cell apoptosis by targeting the PTEN/AKT/eNOS axis. These findings suggest that interrupting the miR-21/PI3K/Akt/eNOS axis could be a new therapeutic strategy for myocardial ischemia/reperfusion injury [25]. PTEN activates the transcriptional program when a large number of microRNAs are silenced. These microRNAs are crucial for maintaining disease states such as cell proliferation, apoptosis, invasion, and adhesion. Additionally, miR-21 targets PTEN and regulates cardiomyocyte apoptosis [24]. The upregulation of PTEN may inhibit the PI3K/Akt signaling pathway, which is related to cell apoptosis. According to the Kyoto Encyclopedia of Genomes (KEGG) pathway analysis of the predicted target genes identified in this study, miR-30a-5p may regulate the PTEN/PI3K/Akt signaling pathway and affect cell apoptosis. It should be noted that changes in PTEN levels during treatment can partially contribute to the survival or death of cells. The inhibition of PTEN and activation of phosphorylated AKT may play a significant role in maintaining the protective properties of myocardial cells.

The Bad protein is phosphorylated by AKT at the Ser residue, causing it to dissociate from Bcl2 or Bcl Extra. Bad then binds to chaperone proteins, resulting in the upregulation of antiapoptotic factors such as Bcl2, A1, and the X-collateral apoptosis inhibitory proteins. This leads to the upregulation of Survivin and the loss of proapoptotic effects [26]. Increased expression of miR-30a-5p led to the suppression of PTEN expression and activation of the PI3K/Akt pathway. Conversely, the silencing of PTEN further augmented the activation of the PI3K/p-AKT pathway. Our study revealed that the miR-30a-5p/PTEN axis increased the expression of the apoptotic proteins Bax and cleaved caspase3 while decreasing the expression of the antiapoptotic protein Bcl-2 and phosphorylated AKT. In H/R-induced h9c2 cells, the downregulation of PTEN by miR-30a-5p resulted in the downregulation of apoptotic proteins and the upregulation of phosphorylated AKT. MiR-22 inhibits myocardial fibrosis in rats with myocardial infarction by targeting the PTEN/Akt/mTOR signaling pathway [27]. Similarly, silencing PTEN with a specific siRNA resulted in a significant increase in phosphorylated Akt compared to that in endothelial cells treated with TNF-α alone [28]. In contrast, overexpression of wild-type PTEN downregulates phosphatidylinositol 3-kinase signaling and upregulates apoptotic proteins in cultured endothelial cells [29]. These studies from other groups support the validity of our investigation.

The role of AKT signaling in inducing or promoting inflammation has been well established. [30]. ApoM is a major carrier of sphingosine 1-phosphate (S1P) and inhibits ox-LDL-induced cellular inflammation by inducing Akt phosphorylation and preventing nuclear translocation of nuclear factor-κB (NF-κB) [31]. Our findings indicate that PTEN knockdown prevented H/R-induced inflammation by promoting AKT activation, which is consistent with previous studies. However, the precise mechanism of downstream genes has not been investigated. This study has several limitations that will be addressed in future research.

## Conclusion

In conclusion, we found that miR-30a-5p downregulates PTEN, leading to the rephosphorylation of AKT, and upregulates apoptotic signaling and inflammatory factor secretion by downregulating AKT. These findings enhance our understanding of the role and mechanisms of miR-30a-5p in the progression of AMI.

### Supplementary Information

**Supplementary Material 1.**

## Data Availability

The data that support the findings of this study are available from the corresponding author upon reasonable request. This study examined publicly accessible datasets. The information is accessible through GEO (https://www.ncbi.nlm.nih.gov/geo/).
