# Supplementary material for: miR-30a-5p attenuates hypoxia/reoxygenation-induced cardiomyocyte apoptosis by regulating PTEN protein expression and activating PI3K/Akt signaling pathway
Source: BMC Cardiovasc Disord. 2024 May 5;24:236. doi: 10.1186/s12872-024-03900-4 (PMC11070099; doi:10.1186/s12872-024-03900-4)
Supplement: Supplementary file 1 — Supplementary Material 1. [file 12872_2024_3900_MOESM1_ESM.docx]

**miR-30a-5p promotes hypoxia-induced inflammation and apoptosis via inhibition of PTEN expression and activation of the PI3K/Akt signaling pathway in cardiomyocytes**

***Supplementary Information***

**Supplementary Methods and Materials**

**Inclusion and exclusion criteria**

Inclusion and exclusion criteria

For individuals in the AMI group, participants were required to meet the following criteria: (1) Post-pectoral pain for over 30 minutes; (2) the presence of the typical evidence of myocardial ischemia on ECG; (3) up-regulation of cTnI and CK-MB; (4) no relief of ongoing chest distress after taking nitrates; (5) the hospital entry in the 12 hours after the occurrence of the chest pain; and (6) patients with the initial onset of AMI. The exclusion criteria were as follows. (1) previous myocardial infarction; (2) previous use of thrombolytic agents for myocardial infarction; (3) severe viral myocarditis; (4) without ahistory of stroke for at least the past 6 months; and (5) known bleeding disorders. Patients were excluded if chest pain was considered to becaused by trauma, medication, and medical intervention. (6) other cancer or related immune

diseases history.

Healthy control subjects were recruited from healthy examination subjects.

Exclusion criteria include a history of head trauma, major diseases, and drug abuse.

**Cell culture**

HT22 cells were purchased from SUNNCELL (SNL-029, SUNNCELL, Wuhan, China). In a 37 °C incubator with 5% CO2, H9c2 cardiomyocytes were grown in DMEM high glucose media with 10% fetal bovine serum and 1% penicillin‒streptomycin. The culture medium was changed and passaged regularly, and the cell status was checked

every other day

**Real-time quantitative PCR (qRT- PCR)**

The reverse transcription process was divided into two steps: the first step was the

genomic DNA reaction, which involved 1 μG RNA, 1 μ DNA, and DEPC water,

totaling 10 μ Mix, and incubation at 45 ℃ for 5 minutes. After the reaction, the reaction was allowed to stand on ice. The second step was a reverse transcription reaction, which involved placing 10 μl of Add 10 to the mixture μ 2 * NovoScript for l ® Gently mix Plus 1st Strand cDNA Synthesis Supermix. The reaction was incubated at 50 ℃ for 30 minutes and then at 75 ℃ for 5 minutes. The reaction was terminated, and the obtained cDNA sample was stored at -20 ℃. The qPCR system included 1 primer before and 1 primer after each μl, 5 μl of subgreen, 2 μl of DEPC water, and 1 μl of cDNA for a total of 10 μl. The amplification conditions were 95 ℃ for 1 minute, with each cycle lasting for 20 seconds at 95 ℃, 1 minute at 60 ℃, and 45 cycles. Using U6 as the internal

reference, each group of target genes and internal reference were repeated for 3 wells.

**Supplemental table**

-

**Table S1 Comparison of clinical data between AMI group and HC group (**x**±s)**

| Parameters | AMI Group  (n = 155) | Healthy Controls  group (n = 90) | *P*-value |
| --- | --- | --- | --- |
| Age ，(years) | 56.58±15.19 | 54.96±15.04 | 0.456 |
| Gener |  |  | 0.1729 |
| Male | 106 | 53 |  |
| Female | 49 | 37 |  |
| BMI： (kg/m2) |  |  |  |
| Smoking: *n* (%) | 94-61 | 56-34 | 0.9138 |
| Drinking: *n* (%)  Diabetes Mellitus: n | 81-74 | 46-44 | 0.9676 |
| Hypertension | 84-71 | 60-30 | 0.07549 |

**Table S2** The information on Antibodies and ELISA Kits.

| **Antibody** | **Vendor** | **Catalog Number** |
| --- | --- | --- |
| PTEN | Cell Signaling Technology | #9188 |

| PI3K | Cell Signaling Technology | #4257 |
| --- | --- | --- |
| p-Akt | Cell Signaling Technology | #4060 |
| Bcl2 | Proteintech Group | 26593- 1-AP |
| Bax | Proteintech Group | 50599-2-Ig |
| iNOS | Proteintech Group | 18985- 1-AP |
| TNF-α | Elabscience | E-EL-M3063 |
| IL-6 | Elabscience | E-EL-M0044c |
| IL- 1β | Elabscience | E-EL-M0037c |

**Table S3** Target genes and datasets intersection genes of miR-30a-5p

| Mirna id | target-gene | target-gene | target-gene | target-gene | target-gene |
| --- | --- | --- | --- | --- | --- |
| miR-30a-5p | ATF1 | KMT2A | MAST4 | EIF5A2 | SEH1L |
| miR-30a-5p | RPA1 | MBNL2 | MIB1 | FBXL14 | SIKE1 |
| miR-30a-5p | CBX3 | OXR1 | MSANTD4 | G3BP1 | SLC30A4 |
| miR-30a-5p | PTEN | PTPN13 | NFATC3 | ICK | TRIM13 |
| miR-30a-5p | CDC37L1 | S100PBP | PBRM1 | INPP4A | YPEL2 |
| miR-30a-5p | PHTF2 | SRSF10 | PCGF3 | KLF12 | ZBTB38 |
| miR-30a-5p | PPARGC1B | TASP1 | PRKAA1 | LYPLAL1 | ZCCHC14 |
| miR-30a-5p | GLCCI1 | TMEM181 | SACS | MFSD6 | ZNF518A |
| miR-30a-5p | KLHL28 | ZBTB6 | SCYL3 | NAA25 | FBXL17 |
| miR-30a-5p | MSI2 | ZSCAN29 | SON | NFYB | AKMIP2 |
| miR-30a-5p | PDE7A | ATG12 | TIA1 | NRIP1 | LARP4 |
| miR-30a-5p | SDAD1 | CDK12 | TNRC6B | OSBPL3 | CEP41 |
| miR-30a-5p | SP4 | DENND1B | TNRC6C | PDP2 | RECK |
| miR-30a-5p | VKORC1L1 | FANCF | USP37 | PLA2G12A | SAMHD1 |
| miR-30a-5p | BTBD7 | FKBP3 | ZNF264 | PREPL | FBXL17 |
| miR-30a-5p | CAND1 | FRS2 | ADAM12 | RAB22A | LMLN |
| miR-30a-5p | CCDC18 | FYCO1 | AGO3 | RAP2B |  |
| miR-30a-5p | EPG5 | IKZF2 | ATG2B | RBM26 |  |


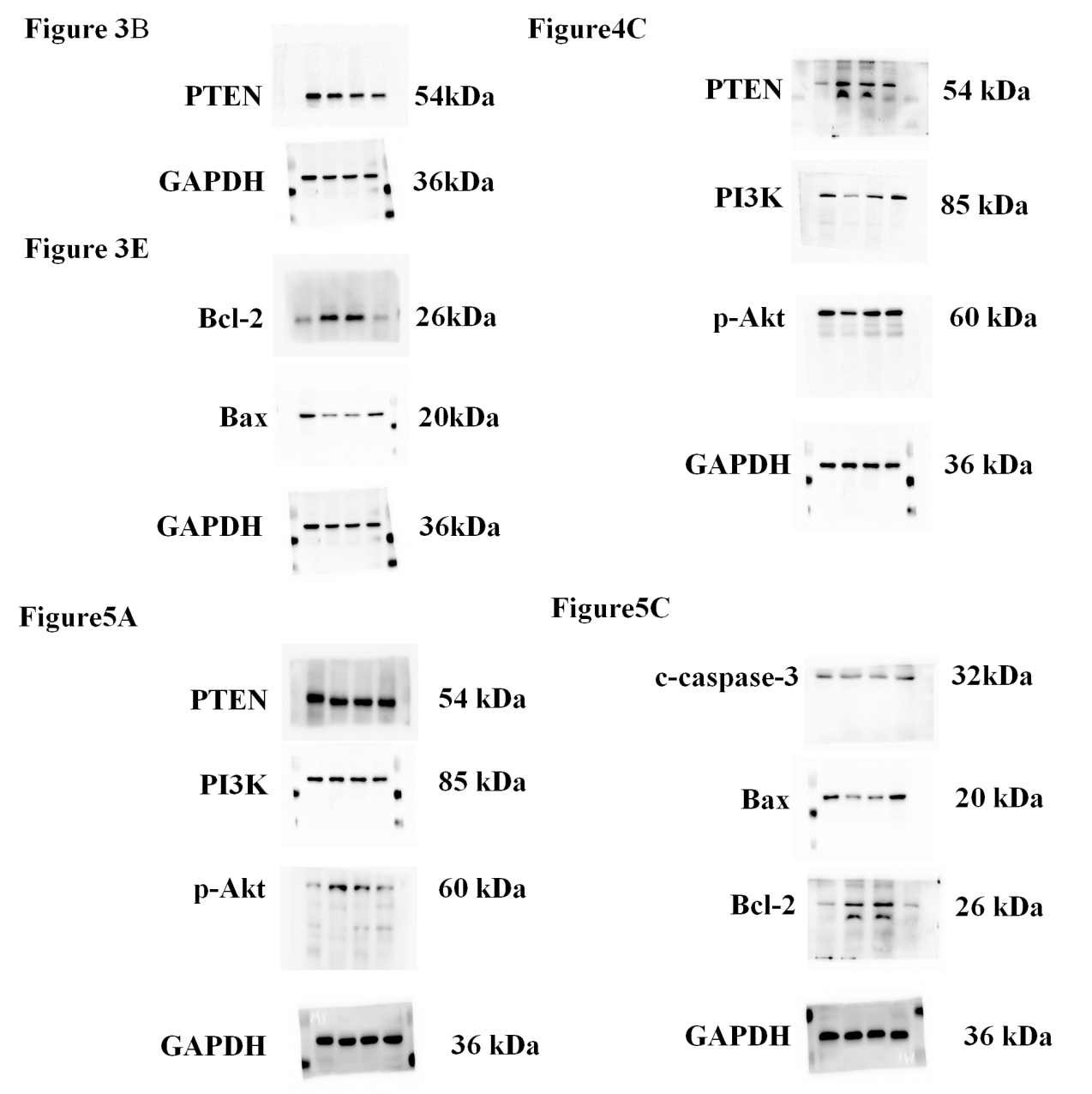


**Supplemental Figure S1**. The original gel/blot images of each figure.
